# Burn Center Management of Severe Necrotic Cutaneous Polyarteritis Nodosa in a Patient With a History of Thymoma

**DOI:** 10.7759/cureus.13134

**Published:** 2021-02-04

**Authors:** Mya Abousy, Angel Byrd, Farah Succaria, Michelle Kerns, Julie Caffrey

**Affiliations:** 1 Department of Plastic and Reconstructive Surgery, Johns Hopkins University School of Medicine, Baltimore, USA; 2 Department of Dermatology, Johns Hopkins University School of Medicine, Baltimore, USA; 3 Department of Dermatology, Howard University Hospital, Washington, DC, USA

**Keywords:** polyarteritis nodosa, cutaneous polyarteritis nodosa, cpan, autoimmune, gangrene, thymoma, ulceration, necrosis

## Abstract

Cutaneous polyarteritis nodosa (cPAN) is a rare, necrotizing vasculitis involving the small- and medium-sized vessels of the dermis and subcutaneous tissues. We report a severe case of cPAN in a patient with an atypical presentation of extensive bilateral lower extremity ulcerations with full-thickness necrosis managed at a burn center. A 70-year-old female with a three-month history of necrotizing cPAN to the bilateral lower extremities underwent surgical excision and autografting at an outside hospital. Postoperatively, she had total graft loss and was begun on prednisone. In the outpatient setting, she was tapered off prednisone and subsequently began to experience an acceleration of the disease process. She was then transferred to our regional burn center for bilateral escharotomy and management of her non-healing, tender, necrotic wounds with distinctive black-brown eschar. One year later, the patient’s wounds continue to decrease in size and heal with her daily regimen of 15 mg of prednisone, 50 mg of cyclophosphamide, and topical silver sulfadiazine application. With the increasing volume of non-burn wound admissions to burn centers primarily of dermatologic etiology, it becomes crucial for burn specialists to familiarize themselves with severe presentations of vasculitides, including cPAN. Further research is necessary to understand the atypical manifestations of this disease for more timely diagnosis and treatment.

## Introduction

Cutaneous polyarteritis nodosa (cPAN) is a rare, necrotizing vasculitis that involves the small- and medium-sized vessels of the dermis and subcutaneous tissues. The classic clinical presentation of this disease is small (0.5-2 cm) subcutaneous nodules (80% of cases in a study of 79 patients), livedo reticularis (56%), and skin ulcers (49%) [1]. Other reported findings included palpable purpura and petechiae [2]. However, cutaneous regions of black-brown necrosis requiring surgical intervention are much less frequent in this patient population and are rarely mentioned in cPAN literature, leading cPAN to often be incorrectly diagnosed as different dermatologic or rheumatologic conditions such as panniculitis, microscopic polyangiitis, or cryoglobulinemic vasculitis [1-4]. Furthermore, as the etiology of this disease is not fully understood, current literature lacks evidence in support of a relationship between autoimmune diseases, such as thymoma, and cPAN.

Although dermatologists typically manage patients with cPAN, our experience with this extreme, gangrenous case of cPAN suggests that a multidisciplinary team approach that includes both dermatologists and burn specialists, such as one readily available in burn centers, may be beneficial for patient outcomes and recovery in such severe cases. With the increasing volume of non-burn wound admissions to burn centers primarily due to dermatologic and rheumatologic cases, it becomes crucial for burn specialists to familiarize themselves with severe presentations of vasculitides [5]. In fact, a 10-year retrospective review conducted in 2010 revealed a 244.9% increase in non-burn admissions to five regional academic burn centers [6]. While the most common non-burn admissions from this study were necrotizing fasciitis (13.7% of admissions) and Stevens-Johnson syndrome/TEN (8.1%), other commonly reported diagnoses included ulcers and livedo reticularis, both of which are manifestations of cPAN [6]. Burn centers have evolved in the past 50 years to include an amalgam of specialists including physicians, nurses, pharmacists, social workers, and physical and occupational therapists that are specialized in the unique requirements of patients with burns and/or severe skin conditions [6]. We report a severe case of cPAN in a patient with a history of thymoma following thymectomy and an atypical presentation of extensive bilateral lower extremity ulcerations with full-thickness necrosis successfully managed at a burn center.

## Case presentation

**Figure 1:**
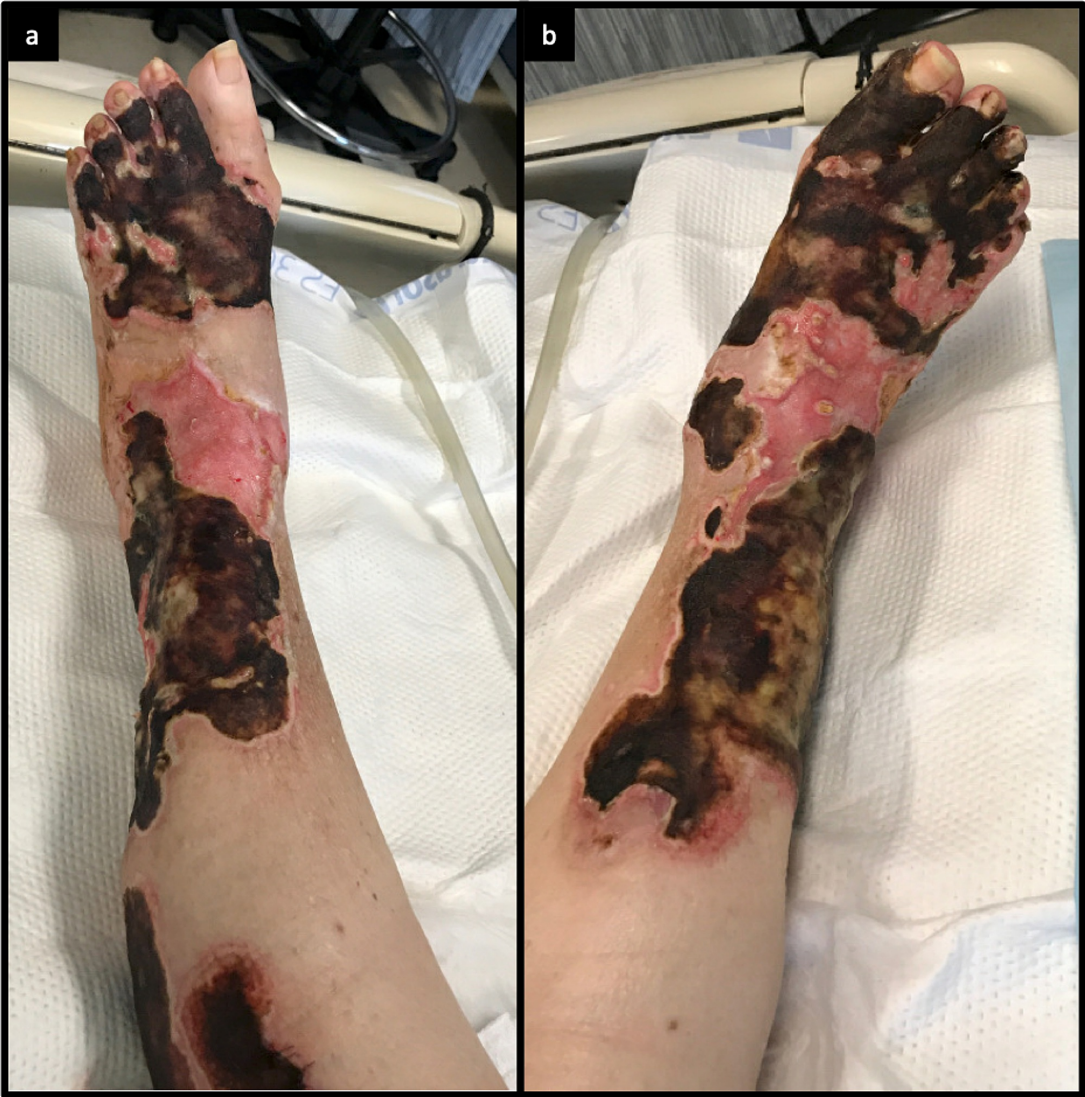
Bilateral (a) right and (b) left lower extremity necrotic, ulcerating wounds with black-brown eschar.

A 70-year-old female presented to an outside hospital with a chief concern of ulcerations of unclear etiology to the bilateral lower extremities for three months. These ulcerations initially began as a small area of erythema on the right ankle that progressed to a blister and subsequent skin erosions. She underwent surgical excision and autografting at this hospital to address these concerns. Postoperatively, she had total graft loss and was begun on prednisone. In the outpatient setting, she was tapered off of her prednisone regimen and subsequently began to experience an acceleration of the disease process. She was then transferred to our regional burn center Intensive Care Unit for management of her non-healing wounds. Upon arrival at our burn center, she required bilateral escharotomy for her necrotic wounds. Her physical examination revealed tender, gangrenous wounds with a distinctive black-brown eschar (Figures 1a, 1b).

**Figure 2:**
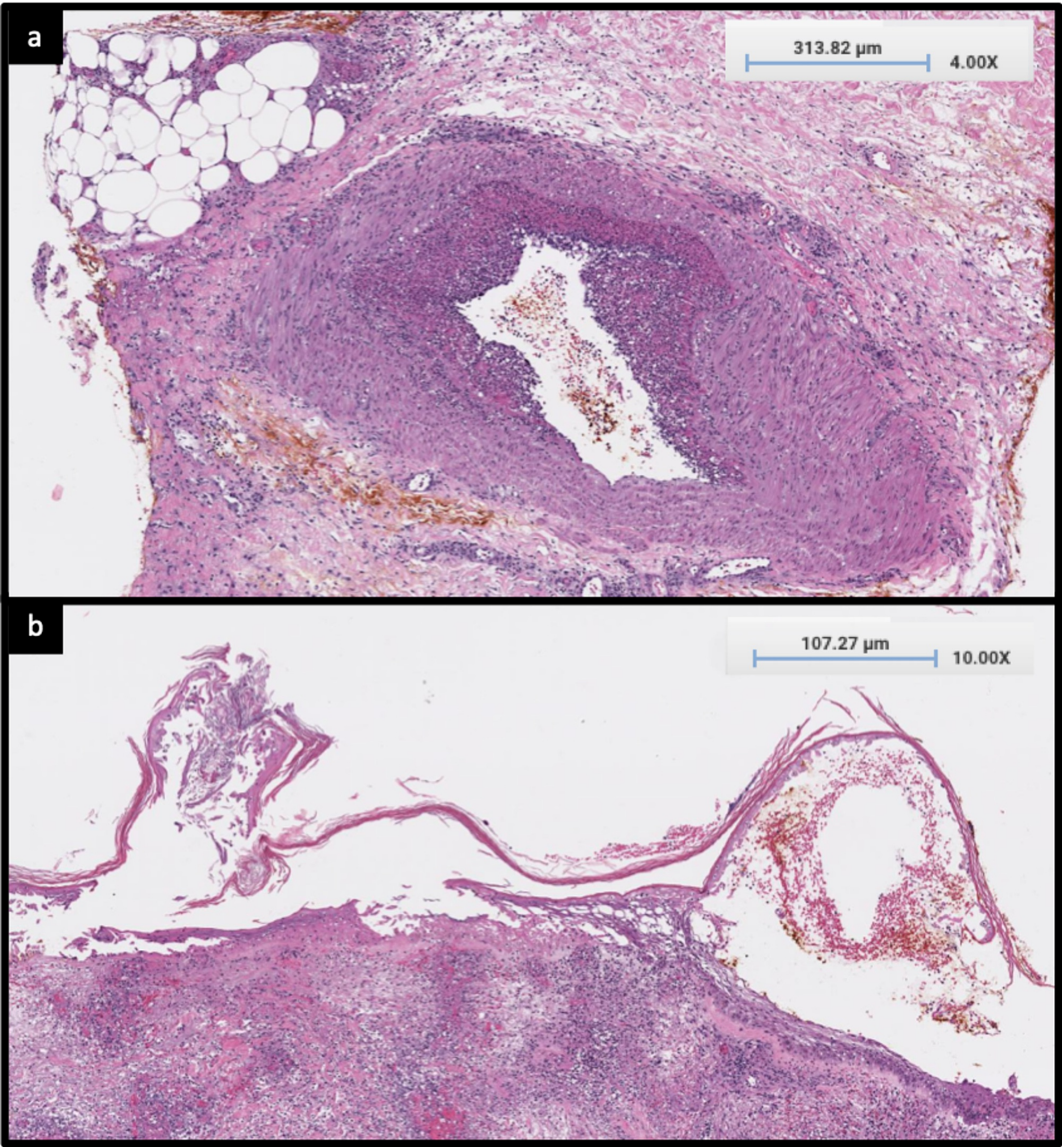
(a) H&E stain, x4 magnification, necrotizing vasculitis involving all the layers of the vascular walls of the medium and small-sized arteries. (b) H&E stain, x4 magnification, overlying necrotic epidermis. H&E: Hematoxylin and Eosin

The lower wounds were biopsied, revealing necrotizing vasculitis involving all layers of the vascular walls of the small and medium-sized arteries, exuberant fibrinoid degeneration of the vessel walls, dense perivascular and interstitial neutrophilic inflammation with neutrophilic debris and extravagated erythrocytes, and edema with an overlying necrotic epidermis (Figures 2a, 2b).

Her past medical history was significant for hypertension, high cholesterol, papillary thyroid cancer status post total thyroidectomy and thymoma status post resection two months before the development of the wounds to her bilateral lower extremities. Serum protein electrophoresis was consistent with hypogammaglobulinemia and acute inflammation, and her IgG levels were markedly low (489 mg/dL; normal: 751-1,560 mg/dL). Her antinuclear antibody (ANA) titers were elevated (1:160; normal: less than 1:40), but perinuclear anti-neutrophil cytoplasmic antibodies (pANCA), cytoplasmic antineutrophil antibodies (cANCA), rheumatoid factor, anti-Ro, myeloperoxidase (MPO), C3 and C4, anti-cardiolipins, and serologies for human immunodeficiency virus, herpes simplex virus, and hepatitis B and C were all within normal limits or negative. The patient did not have any recent viral infections, and testing for Mycobacterium tuberculosis, staphylococcal and streptococcal infections were all negative.

This patient was hospitalized for a total of 19 days at our burn center. Today, she continues to follow-up with our burn center to evaluate her response to her daily regimen of 15 mg of prednisone, 50 mg of cyclophosphamide, and topical silver sulfadiazine application. The patient cannot tolerate a dose lower than 10 mg of prednisone daily due to the exacerbation of her painful wounds. One year later, however, her wounds have significantly decreased in size and continue to heal but have moderate drainage with no signs of infection or cellulitis.

## Discussion

cPAN was first described in 1931 as a distinct version of polyarteritis nodosa (PAN) that presented with similar clinical and histological cutaneous findings but lacked systemic manifestations [7]. It is considered an even more rare disease than systemic PAN, which has an estimated prevalence of 31 cases per million [8]. Although cPAN typically has a more prolonged course than PAN, its prognosis is significantly more favorable than PAN [8]. The etiology of cPAN is still unknown, although recent studies point to the hypothesis of an immune-mediated response that activates the complement pathway resulting in complement deposition in vessels [2,9,10]. Autoimmune hepatitis, for example, consists of immune complex formation and has been associated with cPAN, supporting the hypothesis that cPAN may be an autoimmune-linked disorder [11]. Other case reports suggest a relationship between streptococcal, staphylococcal, hepatitis B/C, or mycobacterium tuberculosis infection and cPAN onset, all of which were absent in our patient [2].

The clinical signs and symptoms of cPAN predominantly include subcutaneous nodules and livedo affecting the legs, with females being affected more commonly than males [12]. While ulcerations and necrosis have been documented in the clinical presentation of cPAN, it is far less common than the usual presentation of small, subcutaneous nodules [12]. This patient’s presentation of expansive necrotizing, black-brown painful ulcerations of impressive size distinguish this case as a severe and atypical manifestation of cPAN. The severity of these lesions is further demonstrated by their resistance to a steroid taper, leading the patient to indefinitely remain on prednisone and cyclophosphamide.

Histopathology of cPAN demonstrates a fibrinoid, necrotizing vasculitis of the small- and medium-sized vessels [12]. Laboratory findings are non-specific, with increased inflammatory markers and a positive ANA titer often documented in previously reported cases [12]. Unfortunately, there are no widely accepted and validated diagnostic criteria for cPAN, making the diagnosis primarily one of exclusion. However, Furukawa et al. drafted a set of diagnostic criteria in 2012: (1) aforementioned cutaneous manifestations and histopathological findings and (2) an absence of systemic symptoms including arthralgias, myalgias, and/or peripheral neuropathy in regions without skin manifestations, fever, hypertension, or any internal organ complications [12]. cPAN treatment options have yet to be validated by prospective studies, although retrospective studies have endorsed the use of systemic steroids and even immunosuppressants for patients who do not respond to steroids alone [1,12]. Non-steroidal anti-inflammatory agents are another treatment option, yet these medications have historically shown variable efficacy in disease progression [1,12].

From an immunologic perspective, the patient presented following a thymectomy due to thymoma. There is a well-established relationship between thymoma and autoimmune disease, though the exact mechanism of autoimmunity is still debated [13]. Some studies suggest that thymomas introduce autoantigen-specific T cells that evade the induction of self-tolerance in the thymic medulla, thus causing immune dysregulation. Other literature proposes that genetic modifications in neoplastic thymoma cells create dysfunctional T cells that are self-reactive [13]. However, similar to this patient’s presentation, thymectomy has been found to precede the development of autoimmune disease [13]. This challenges current hypotheses of autoimmune disease etiology in patients with thymoma, as thymectomy should theoretically eliminate the source of the immune dysregulation. Regardless of the unclear cause of autoimmune disease in the presence of thymoma, there is evidence to support that these two conditions may be correlated, suggesting a potential relationship between this patient’s development of cPAN and the immune dysregulation induced by her thymoma [2,9-11,13].

Despite generally favorable prognosis in patients with cPAN, close follow-up is necessary to monitor recurrence as well as potential progression to systemic disease. The majority of the current literature suggests that cPAN does not progress to PAN, and there is only one case report to date that identifies two patients who solely had cutaneous manifestations for over a decade before later progressing to systemic PAN [1,7,14]. Patients with recurrent skin ulcers or immunologic abnormalities such as positive ANA titers, RF levels, or elevated erythrocyte sedimentation rate may require more frequent monitoring of cardiac, renal, gastrointestinal, and neurologic function for signs of disease progression [12]. Periodic testing of creatinine levels may help detect asymptomatic kidney disease as an early sign of disease progression. As this patient has been using steroids for a prolonged period of time, it is essential to monitor for consequences of long-term steroid use including osteoporosis, Cushing’s syndrome, dyslipidemia, cataracts, and glaucoma.

This patient’s care ultimately depended on a multidisciplinary approach from her management team. Throughout her stay at our hospital, she received repetitive care from dermatologists, rheumatologists, physical therapists, social workers, and dieticians in addition to burn specialists. Of note, the dermatology team was crucial in diagnosing this patient with cPAN through dermatopathological evaluation and in providing pertinent management recommendations, such as transitioning to chronic immunosuppression and assessing whether repeat biopsies were necessary when lesions worsened. Rheumatology further assisted in the diagnostic process given the complexity of this patient’s presentation and helped determine which immunosuppressants would best benefit this patient.

## Conclusions

This report describes an extreme case of a patient with cPAN who ultimately required treatment at a burn center to manage her non-healing, ulcerative wounds that have substantially improved to date with a combination of prednisone, cyclophosphamide, and topical silver sulfadiazine. The patient’s case illustrates one of the many ways in which this rare and poorly understood vasculitis may manifest and provides an example of disease management. Isolated gangrenous necrosis with no systemic symptoms, while an uncommon manifestation of cPAN, should lead practitioners to include this disease on the differential and to even consider treatment in a burn center where patient care can be specialized and multifaceted.

Conditions treated at burn centers are often stigmatizing given their visually noticeable manifestations that may negatively impact self-esteem, leaving patients to carry a major psychosocial burden both during their hospital stay as well as upon discharge. The multidisciplinary approach to patient care in modern-day burn centers is thus well-equipped to address these specific patient needs given the variety of training backgrounds from team members. Between intensive wound care, infection control, physical and occupational therapy, social work, and immediate availability for surgical care, burn centers have evolved to handle a variety of wounds of non-burn etiology, including severe vasculitides. While cPAN has historically been managed by dermatologic teams, we recommend a multidisciplinary approach to this disease that includes burn specialists, particularly in such extreme cases as this patient’s presentation that ultimately resulted in surgical interventions and aggressive pharmacologic therapy.
